# Effects of Illumination Color on Hypothalamic Appetite-Regulating Gene Expression and Glycolipid Metabolism

**DOI:** 10.3390/nu16244330

**Published:** 2024-12-15

**Authors:** Qi Wang, Qianru Li, Tuo Quan, Hongshan Liang, Jing Li, Kaikai Li, Shuxin Ye, Sijia Zhu, Bin Li

**Affiliations:** 1 College of Food Science and Technology, Huazhong Agricultural University, Wuhan 430070, China; 13523904575@163.com (Q.W.); 18468088557@163.com (Q.L.); quantuo@mail.hzau.edu.cn (T.Q.); lianghongshan@mail.hzau.edu.cn (H.L.); lijingfood@mail.hzau.edu.cn (J.L.); likaikai@mail.hzau.edu.cn (K.L.); m13628691513@163.com (S.Y.); zsj18899590970@163.com (S.Z.); 2 Key Laboratory of Environment Correlative Dietology, Ministry of Education, Huazhong Agricultural University, Wuhan 430070, China

**Keywords:** lighting color, dietary preference, glucolipid metabolism, appetite

## Abstract

Irregular illumination is a newly discovered ambient factor that affects dietary and metabolic processes. However, the effect of the modulation of long-term light exposure on appetite and metabolism remains elusive. Therefore, in this current study, we systematically investigated the effects of up to 8 weeks of exposure to red (RL), green (GL), and white light (WL) environments on appetite, food preferences, and glucose homeostasis in mice on both high-fat and low-fat dietary patterns. It was found that the RL group exacerbated high-fat-induced obesity in mice compared with GL- or WL-treated mice. RL-exposed mice exhibited worsened metabolic profiles, including impaired glucose tolerance/insulin sensitivity, elevated lipid levels, and reduced serum insulin levels. Serological analyses showed that RL exposure resulted in decreased leptin levels and increased levels of orexigenic and hunger hormones in mice. Further qPCR analysis showed that the expression levels of the hypothalamic appetite-related genes NPY and AgRP mRNA were upregulated in RL-treated mice, while the expression level of the appetite suppressor gene POMC mRNA was downregulated. The results of this study will be instructive for the regulation of appetite and metabolism from the perspective of illumination colors.

## 1. Introduction

Sunlight is an essential life energy and the ultimate energy source for modern life. It empowers plant photosynthesis, aids in vitamin D synthesis, and enhances calcium absorption. With the popularization of light-emitting diodes (LEDs), lighting has attracted much attention [1,2]. For numerous light-sensitive mammals, including humans and mice, the illumination environment plays an important role in appetite, dietary preferences, glucolipid metabolism, and physiological biochemistry and behavior [3,4,5]. Appetite reflects the body’s reaction to energy equilibrium and nutritional conditions [6]. Although certain elements of food preferences are inherent, external factors can influence them and motivate individuals to make selections that support their long-term health goals [7,8,9]. Healthy eating is inextricably linked to reducing the risk of chronic disease and obesity and promoting overall health. For instance, high-intensity white light (50 and 150 lux) triggered a stronger glucose response compared with low-intensity white light (5 and 20 lux). Additionally, exposure to green light resulted in glucose intolerance in mice [10]. Studies show that short light cycles impair glucose balance and downregulate genes linked to glucose processing in flounder muscle when compared with standard cycles (12 h). Furthermore, rats subjected to prolonged light cycles exhibited higher blood glucose levels [11]. Consequently, researchers are increasingly focused on how the lighting environment may influence our dietary habits, glycolipid metabolism, and overall physiological functions.

Ambient light information that affects appetite and metabolism depends upon several parameters, including light intensity, photoperiod, light duration, and color [12,13]. For example, exposure to GL leads to increased weight gain, diminished glucose tolerance, familial hyperlipidemia, and hepatic steatosis in obese mice [14,15,16]. Research indicates that blue light can lead to photochemical harm, resulting in oxidative stress, inflammatory apoptosis, and DNA impairment [12,17]. Furthermore, a clinical study revealed that exposure to blue light during morning or evening hours heightened insulin resistance and disrupted metabolic processes [18]. Numerous studies indicate that lighting conditions can affect both behavior and physiological functions in animals [19]. Anayanci et al. discovered that acute exposure to artificial light at night in male Sudan grass rats led to glucose intolerance, reduced insulin secretion, and increased sugar consumption. Although these studies fully supported the role of illumination color in physiological and metabolic regulation, they often involved short-term light exposure or short durations. Consequently, the metabolic effects of extended light exposure remain unknown. Additionally, the impact of light color on appetite has not been thoroughly investigated.

Non-visual imaging in the brain induces light receptivity in the mammalian retina, not only limited to the vision-forming retinal and cone cells but also the expression of the photopigment melanopsin [20]. In varying light conditions, light triggers the nonvisual system of the hypothalamus, leading to diverse physiological changes. The hypothalamus serves as a crucial area for energy management and appetite regulation, with the arcuate nucleus (ARC) significantly involved in processing signals that influence appetite. The NPY/spiny mouse gene-related protein (agouti gene-related protein (AgRP)) and opioid promoter melanocortin (proopiomelanocortin (POMC)) neurons, which are mainly located in the region of the arcuate nucleus, exert not only appetitive stimulatory effects but also inhibit the anorexigenic effects of POMC neurons via Npy-y1 receptors or the neurotransmitter γ-aminobutyric acid (GABA) [21,22,23]. Leptin acts on the hypothalamus through circulation and binds to the neurohumoral skin in the hypothalamus related to energy metabolism, ultimately regulating the animal’s ability to feed and produce heat. The appetinogen gene code is specifically expressed in the lateral hypothalamic nucleus and its neighboring regions, and data have shown that it is involved in the regulation of a variety of physiological functions, including appetite regulation, energy metabolism, and homeostasis.

These findings indicate that light can influence physiological and metabolic processes, but this is mainly based on short-term light exposure. Currently, the impact of prolonged exposure to monochromatic light on feeding behavior and appetite in mice, along with the mechanisms involved, remains largely unexplored. Therefore, we exposed mice to various light colors (RL, GL, and WL) for two months. We employed an ad libitum feeding strategy, offering both a low-fat diet (LFD) and a high-fat diet (HFD) in their cages. This approach aimed to examine how long-term exposure to different lighting conditions affected the mice’s feeding preferences, appetite, and glycolipid metabolism.

## 2. Materials and Methods

### 2.1. Animals

All animal procedures in this investigation conformed to the *Guide for the Care and Use of Laboratory Animals* published by the USA National Institutes of Health, publication no. 85-23, revised in 1996, and provisions were approved by the HCCA Laboratory Animal Care Committee (permit number: HZAUMO-2023-0268).

**Figure 1:**
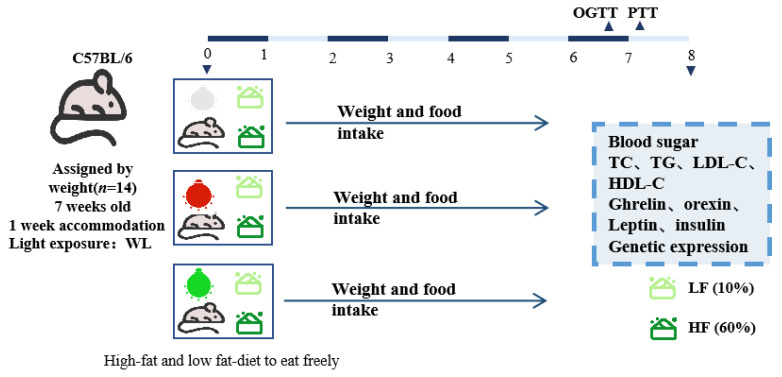
Graphical representation of the experimental design.

Male C57BL/6J mice (7 weeks old) were housed in a standard rearing environment (25 °C; 50% ~60% humidity; 12 h–12 h light–dark cycle) for 1 week. For conditioning, white light (WL) was used. Then, the animals were randomized and divided into three groups (*n* = 14 per group) and subjected to light exposure under different LEDs, RL (peak at 620 nm), GL (peak at 520 nm), or WL (peak at 465 nm), for two months, respectively (Figure 1). The lamps were maintained at 150 lux at all times (12 h–12 h light–dark cycle; time: lights on at 19:00 (ZT19) and lights out at 7:00 h (ZT7)).

To prevent interference from other environmental factors (e.g., natural light and noise), each cage was housed in a customized, light-shaded, sound-insulated, and ventilated integrated cabinet (150 cm × 150 cm × 55 cm). Meanwhile, both low-fat (10% of energy from fat, Reseatch Diets, New Brunswick, NJ, USA) and high-fat (60% of energy from fat, Research Diets, USA) feeds were provided in each group of cages, and the animals were free to choose which they preferred. The specific formulations of the feeds are shown in the table. Body weights were measured every 2–3 days, and dietary intake was measured daily for 8 weeks (3 times/week).

### 2.2. Sample Collection and Processing

After 8 weeks of feeding, all mice fasted for 12 h. Blood was collected from the eyeballs of mice anesthetized with ether on the following day. The blood was collected in 1.5 mL disposable centrifuge tubes and allowed to stand at room temperature for 2 h. Each supernatant was taken and divided after the serum was precipitated (4 °C; 3000 r/min; 10 min). After the blood collection, each mouse had its neck broken and was executed, and then the autopsy was started immediately, and the liver, kidney, hypothalamus, and adipose tissues were removed. Hair and other impurities were washed away with PBS buffer solution. All samples to be tested were stored at −80 °C. The mouse bedding and feed were changed every two days, with simultaneous recording of food intake and body weight. The formulation and nutritional content of the feed for each group of mice are detailed in Table 1.

### 2.3. Oral Glucose Tolerance Test (OGTT) and Pyruvate Tolerance Test (PTT)

An oral glucose tolerance test (OGTT) and a pyruvate tolerance test (PTT) were performed ten days before and five days before the end of the experiment. Before the tests, the mice fasted overnight. On the following day, the mice were excised from the tail tip with a scalpel at about 1 mm, and a hand-held glucometer measured their fasting (t = 0 min) blood glucose concentration. Subsequently, a 20 wt% glucose solution or pyruvate was administered by gavage at a dose of 2 g/kg bw or 1 g/kg, and the blood glucose levels of the mice were examined at t = 15 min, 30 min, 60 min, and 120 min after the end of the gavage [24]. The homeostatic model assessment (HOMA) was adopted to quantify the degree of insulin resistance in the mice. The homeostatic assessment model’s insulin resistance index (HOMA-IR) was calculated using the following formula: HOMA-IR = blood glucose concentration (mmol^−1^) × insulin concentration (mUL^−1^)/22.5. The area under the curve (AUC) was calculated by Graphpad prism 9.5 and statistically analyzed.

### 2.4. Histological Analyses

The epididymal fats and livers were isolated and fixed with 4% paraformaldehyde for 24 h and then embedded in paraffin, cut into 4 μm sections, and stained with hematoxylin–eosin (H&E) for morphological observation. The morphology was observed under an Olympus light microscope (Japan).

### 2.5. Serological Analysis

Blood samples were collected in non-heparinized tubes and centrifuged at 4 °C at 4000× *g* for 10 min. Serum triglyceride (TG), total cholesterol (TC), low-density lipoprotein cholesterol (LDL-C), high-density lipoprotein cholesterol (HDL-C), alanine aminotransferase (ALT), and aspartate aminotransferase (AST) levels were detected by a commercial kit (Nanjing Jiancheng Biotechnology Institute). The levels of ghrelin, orexin, leptin, insulin, POMC, and NPY were determined by an ELISA kit.

### 2.6. Hypothalamic Gene Expression

The mRNA expressions of leptin, orexin, NPY, AgRP, and POMC in the mouse hypothalamus were determined by fluorescence quantitative PCR. The hypothalamic tissue was fully ground by taking about 20 mg of ice and placing it in 1 mL of pre-cooled TRIpure. The homogenate was carefully poured into a 1.5 mL EP tube and fully mixed with 250 μL of trichloromethane. After standing on ice for 5 min, the hypothalamic tissue was centrifuged at 10,000× *g* at 4 °C for 10 min. An amount of 500 μL of the supernatant was placed into a 1.5 mL EP tube, and the same volume of isopropyl alcohol precooled at 4 °C was added and mixed well, and the mixture was left to stand at −20 °C for 15 min. The RNA was cleaned, precipitated several times, and dried on a super-clean worktable. After the ethanol was fully volatilized, 10 μL of RNase-Free Water was added to fully dissolve the RNA.

The first cDNA was synthesized by an EntiLink™ 1st Strand cDNA synthesis kit. The reaction liquid was prepared on ice. After the reaction at 70 °C for 5 min in the PCR instrument, it was quickly placed on ice to cool for 2 min and then kept at 37 °C for 60 min in the PCR instrument. The reaction liquid was removed, treated at 85 °C for 5 min, and stored at 4 °C for later use. An EnTurbo™ SYBR Green PCR SuperMix kit and a StepOne™ Real-Time PCR instrument were used for real-time fluorescence quantitative PCR analysis. After predenaturation at 95 °C for 3 min, denaturation was conducted at the same temperature for 10 s, followed by annealing at 58 °C for 30 s, extension at 72 °C, and reading plate analysis for 30 s for 40 cycles. Referring to the study by Livak et al., the relative mRNA expression was calculated by the ΔΔCT method [25]. Table 2 lists the primers of the targets tested.

### 2.7. Statistical Analysis

Unless otherwise specified, all results are expressed as means ± standard deviation. Graphpad Prism 9.5 was used for drawing, and IBM SPSS Statistics 26 was used for statistical analysis. Before performing ANOVA, all data were quality-checked using boxplot analysis to guarantee they followed normal distributions. The one-way ANOVA method was used to evaluate the treatment effects of each outcome, and Fisher’s LSD method was selected for post hoc analysis to compare the means between groups. The difference was significant at *p* < 0.05. The following parameters were calculated based on the food intake of each mouse and the energy ratio of each ingredient in the diet [26]:

Total energy intake (kcal) = LFD intake (g) × 3.85 (kcal/g) + HFD intake (g) × 5.24 (kcal/g); carbohydrate energy intake (kcal) = LFD intake (g) × [3.85 × 20.1% (kcal/g)] + HFD intake (g) × [5.24 × 70.0% (kcal/g)]; fat energy intake (kcal) LFD intake (g) × [3.85 × 10% (kcal/g)] + HFD intake (g) × [5.24 × 59.9% (kcal/g)]; protein energy intake (kcal) = LFD intake (g) × [3.85 × 20% (kcal/g)] + HFD intake (g) [5.24 × 20% (kcal/g)].

## 3. Results and Analysis

### 3.1. Changes in Food Intake and Body Weight of Mice

**Figure 2:**
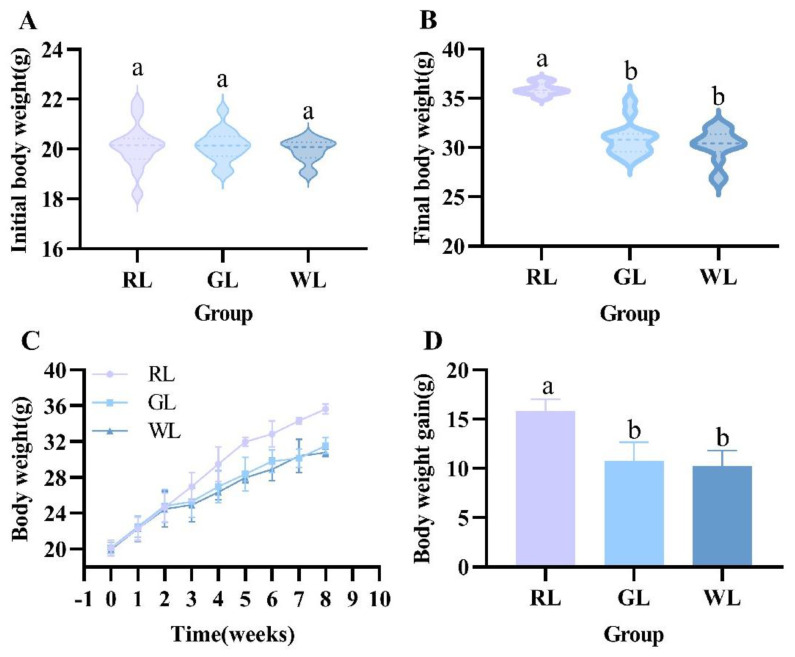
Effect of illumination color on body weight in mice. Initial body weight (**A**), final body weight (**B**), body weight change curve (**C**), and body weight gain (**D**) (*n* = 14). ab represent significant differences between groups at *p* < 0.05 level.

**Figure 3:**
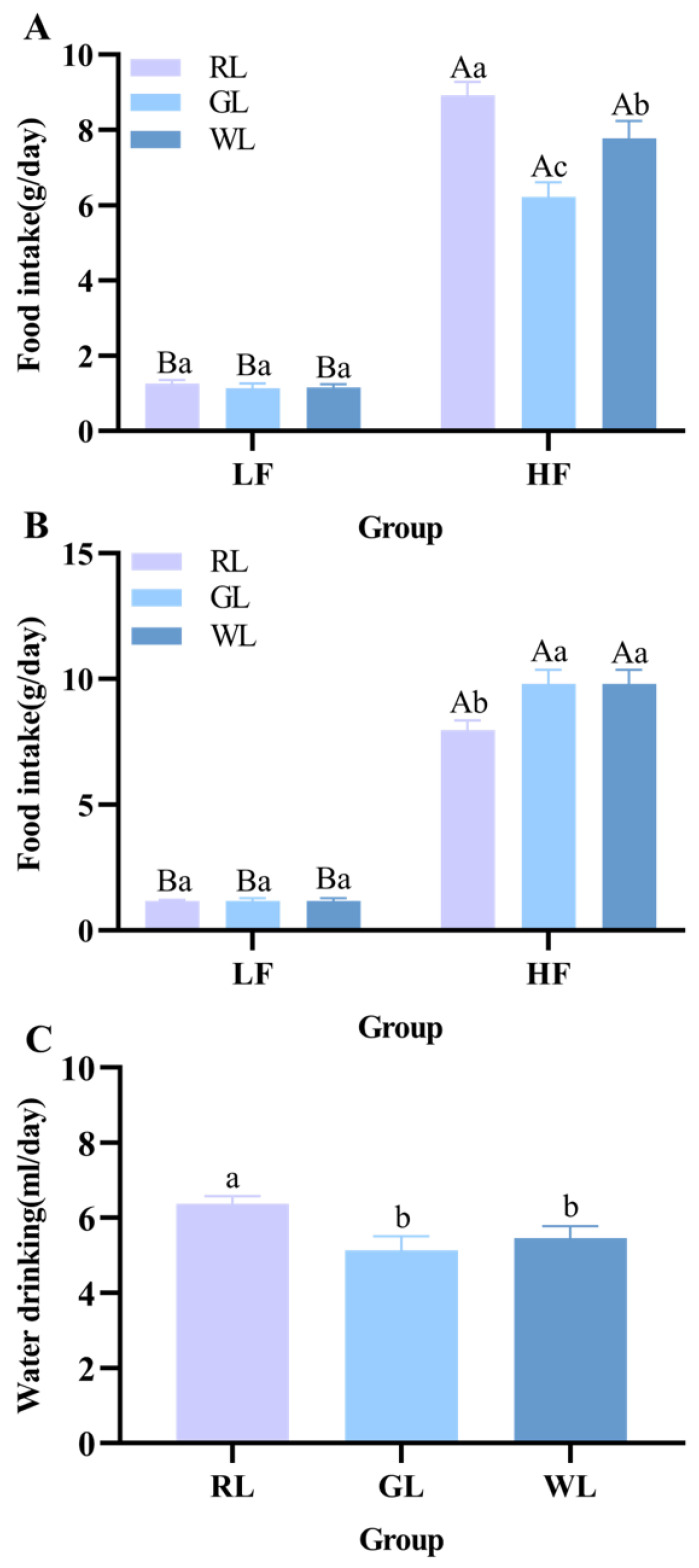
Effects of different illumination colors on food intake in mice. Light period food intake (**A**), non-light period food intake (**B**), and water intake (**C**) (*n* = 14). a–c represent significant differences between groups and A–B represent significant differences within groups at *p* < 0.05 level.

**Figure 4:**
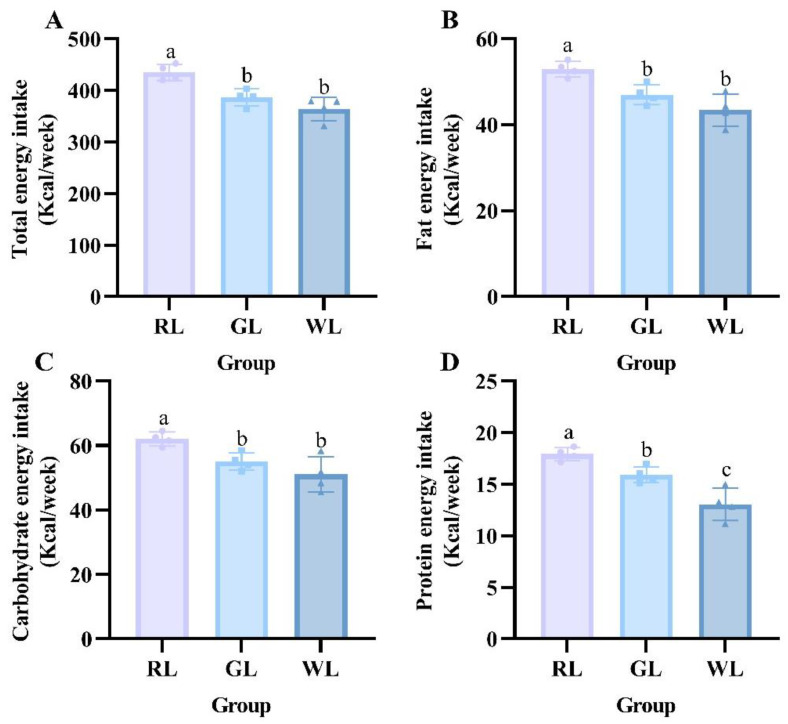
Effects of different light colors on total and macronutrient energy intake in mice. Total energy intake (kcal/week) (**A**), fat energy intake (kcal/week) (**B**), carbohydrate energy intake (kcal/week) (**C**), and protein energy intake (kcal/week) (**D**) (*n* = 14). a–c represent significant differences between groups at *p* < 0.05 level.

Both low-fat and high-fat diets were placed in each cage, and the color of the lighting was changed to explore the effects of the lighting environment on the weight gain and feeding preference of the mice. The results are shown in Figure 2, Figure 3 and Figure 4. After randomization, the weight of the mice in each group was about 20 g, and the formal experiment began. As can be seen from Figure 2C, when the mice were exposed to different colors of light, the weight of the mice in each group showed significant differences (*p* < 0.05). Red lighting made the mice more obese and increased weight gain, while green and white light had less of an effect on weight (*p* < 0.05). By the end of the experiment, the weight of the mice under red light was more than 35 g, which was higher than that of the WL and GL groups (*p* < 0.05) (Figure 2B). Regarding food intake, when both low-fat and high-fat diets were placed in the cage, the lighting color did not affect the choice of food (*p* > 0.05), and the intake of high-fat food under the three lighting colors was much higher than that of low-fat food (*p* < 0.001) (Figure 3A). Under the different lighting colors, there was no significant difference in the intake of the low-fat diet (Figure 3A). The calculation of the energy intake from macronutrients relied on the carbohydrate, fat, and protein calories found in both the low-fat and high-fat foods (*p* > 0.05) (Figure 4). The mice subjected to red illumination displayed a strong preference for foods rich in carbohydrates, fats, and proteins (Figure 4) (*p* < 0.05). As shown in Figure 3A, during the light period, the intake of the high-fat diet in the mice in the RL group was significantly higher than that in the mice under the other two lighting conditions (*p* < 0.05), and a similar trend was observed in the drinking water (Figure 3C). The intake of the high-fat feed in the RL group was significantly lower than that in the WL and GL groups during the non-light period (*p* < 0.05) (Figure 3B). This may indicate that red light promoted the mice’s eating behavior to some extent.

### 3.2. Weight and Histological Observations of Liver and Fat

**Figure 5:**
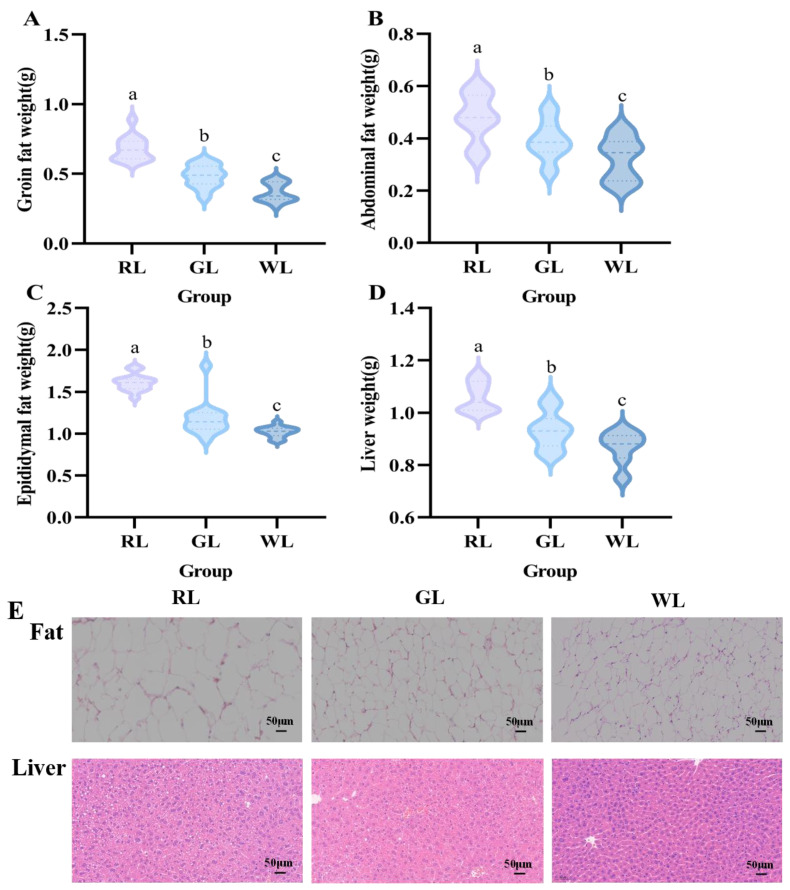
Tissue weights of groin fat (**A**), abdominal fat (**B**), epididymal fat (**C**), and liver (**D**) and representative H&E staining of the adipose and liver tissue (**E**) under different illumination colors (*n* = 14). a–c represent significant differences between groups at *p* < 0.05 level.

After dissecting the mice, the liver, epididymal fat, abdominal fat, and groin fat tissues were isolated and weighed. The long-term high-fat diet promoted liver weight gain and fat accumulation in the mice. As depicted in Figure 5, compared with the WL and GL groups, the weight of the liver, abdominal fat, and epididymal fat in the RL group was significantly increased (*p* < 0.05).

The results of H&E staining revealed that the hepatocyte and adipose tissue morphology in the RL group had large adipose vacuoles, which indicated that long-term red illumination exacerbated the excessive accumulation of intracellular lipids (Figure 5E). The GL and WL groups also had obvious adipose vacuoles, but the overall cellular structure was relatively normal and homogeneous.

### 3.3. Effect of Lighting Color on Glucose Regulation

**Figure 6:**
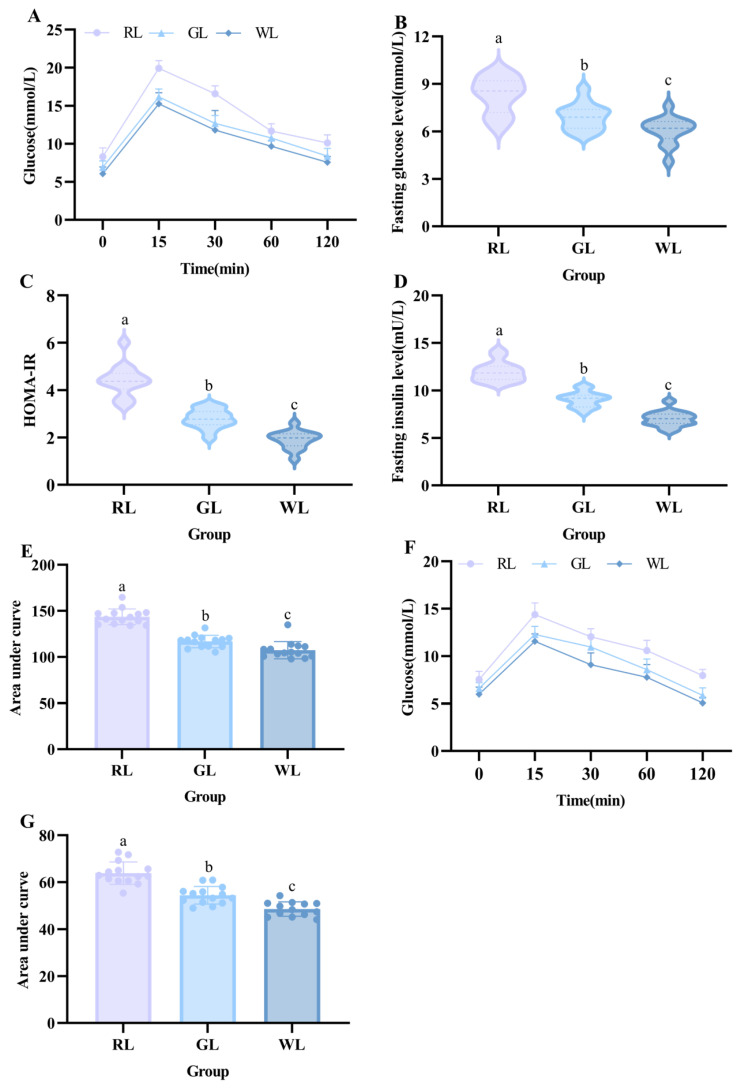
Effects of different illumination colors on glucose homeostasis in mice. OGTT curve (**A**), fasting glucose (**B**), insulin resistance index (**C**), fasting insulin (**D**), OGTT AUC (**E**), PTT curve (**F**), and PTT AUC (**G**) (*n* = 14). a–c represent significant differences between groups at *p* < 0.05 level.

Subsequently, we conducted a tolerance test to evaluate glucose homeostasis insulin sensitivity in the mice. As can be seen from Figure 6A,E, the mice in the RL group showed insulin resistance under long-term lighting conditions with different colors (*p* < 0.05). During the 2 h metabolic process, the blood glucose content in the RL group mice remained high. The blood sugar levels of the mice in each group reached the peak value 15 min after intragathy, and the blood sugar value of the mice in the RL group was much higher than that in the other groups, while that in the GL group was lower than that in the WL group (*p* < 0.05) (Figure 6A). After 2 h of metabolism, the blood glucose level of the mice in the RL group decreased gently and, eventually, was much higher than their baseline blood glucose level, while those of both the GL and WL groups returned to a normal state (*p* < 0.05) (Figure 6A). Figure 6C presents the HOMA-IR of the mice in each group, and insulin resistance was significantly higher in the RL group (*p* < 0.05). The OGTT analysis showed that both RL and GL conditions impaired glucose tolerance in high-fat-fed mice (*p* < 0.05). High-fat feeding caused glucose intolerance, and RL and GL exacerbated this pathology, which is consistent with Zhang’s study [27]. Furthermore, the analysis of the baseline blood glucose and insulin levels of the mice in each group demonstrated that prolonged illumination disturbed the ability of the RL group of mice to stabilize their blood glucose, resulting in significantly higher blood glucose and insulin levels under fasting than those of the WL and GL groups of mice (*p* < 0.05) (Figure 6B). The PTT experiments revealed that the glucose output to the blood of the mice exposed to both RL and GL was higher than that of the WL group and was highest in the RL group (Figure 6F,G) (*p* < 0.05). In conclusion, RL caused the most significant disruption of glucose homeostasis within the body of all the lights tested.

### 3.4. Serum Lipid Metabolism Indexes

**Figure 7:**
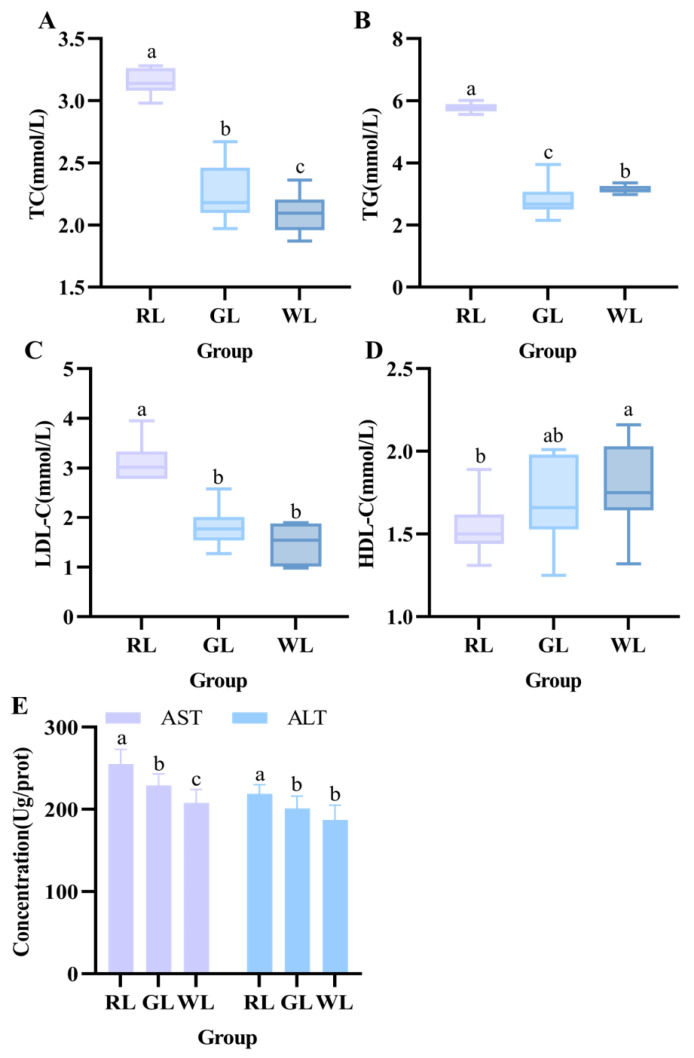
Effects of different illumination colors on circulating lipid levels in mice. TC (**A**), TGs (**B**), LDL-C (**C**), HDL-C (**D**), and AST and ALT (**E**) (*n* = 14). a–c represent significant differences between groups at *p* < 0.05 level.

Excessive intake of high-calorie foods is an important risk factor for hyperlipidemia, which is typically characterized by high levels of TC (total cholesterol), TGs (triglycerides), and LDL-C (low-density lipoprotein cholesterol) in the plasma and low levels of HDL-C (high-density lipoprotein cholesterol). As shown in Figure 7, for the serum lipid profiles, we observed that high-fat feeding under RL caused hyperlipidemia in mice, resulting in severely high levels of TC, TGs, and LDL-C (*p* < 0.05). Compared with the WL group, the plasma levels of TC, TGs, and LDL-C in the RL group were increased by 1.51 times, 1.84 times, and 2.31 times, respectively. The ratio of LDL-C to HDL-C was also enhanced (Table 3). Alanine aminotransferase (ALT) and aspartate aminotransferase (AST) are commonly used as biomarkers of liver injury [28]. Figure 7E illustrates that AST and ALT levels were significantly elevated in mice raised on the high-fat diet. These results reveal that RL adversely affected lipid and liver metabolism parameters.

### 3.5. Effects of Different Lighting Colors on Appetite Hormones

**Figure 8:**
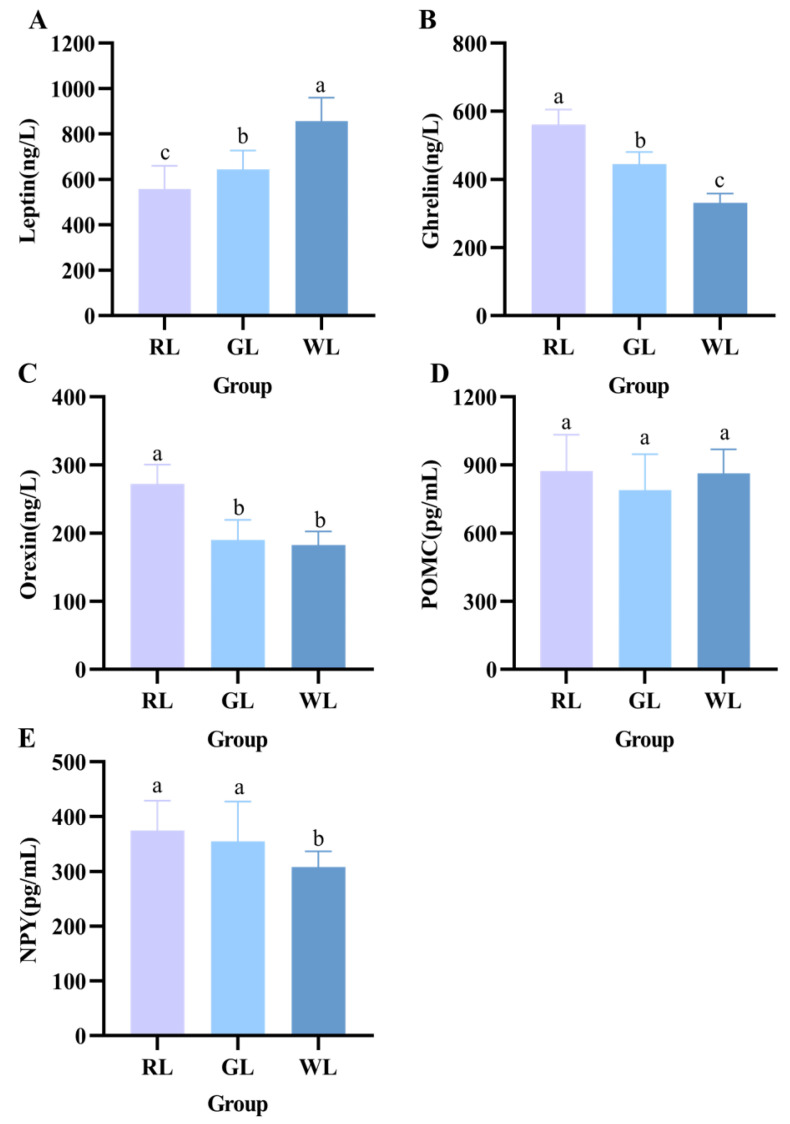
Effects of different illumination colors on appetite hormones in mice. Leptin (**A**), ghrelin (**B**), orexin (**C**), POMC (**D**), and NPY (**E**) levels (*n* = 14). a–c represent significant differences between groups at *p* < 0.05 level.

As shown in Figure 8, we detected the levels of appetite hormones in mice under different lighting conditions. The leptin content in mice under WL was higher than that in mice under RL and WL (*p* < 0.05). Figure 8C and Figure 6D illustrate that mice secreted increased amounts of orexin and insulin when exposed to RL, potentially resulting in elevated ghrelin levels (Figure 8B) (*p* < 0.05). Meanwhile, AGRP levels were enhanced in mice exposed to red light, but there was no significant change in POMC levels in any of the three groups of mice.

### 3.6. Fluorescence Quantitative PCR Analysis

**Figure 9:**
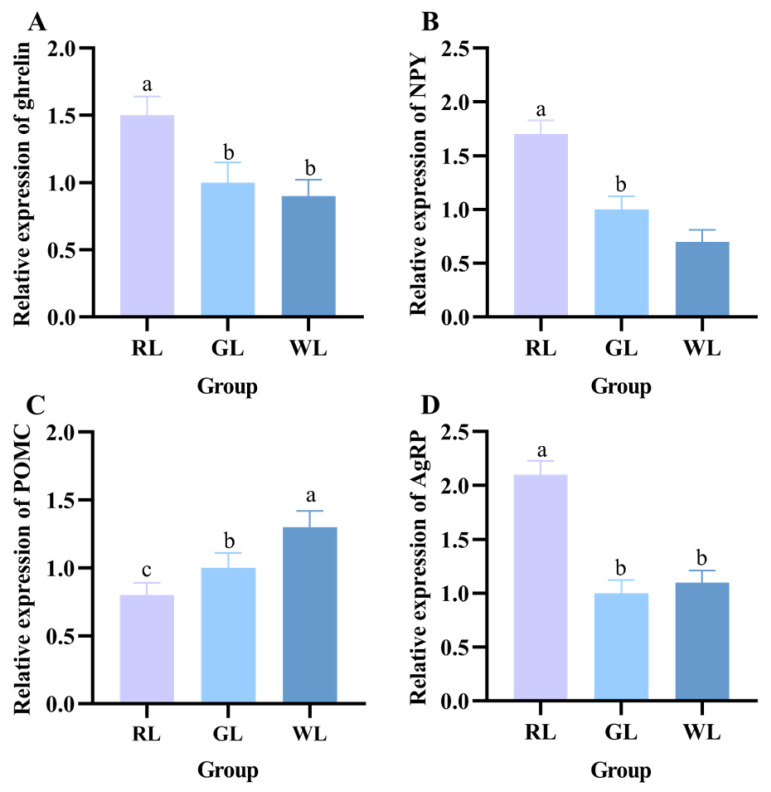
Effects of different lighting colors on the levels of appetite genes in mice. Ghrelin (**A**), NPY (**B**), POMC (**C**), and AgRP (**D**) (*n* = 14). a–c represent significant differences between groups at *p* < 0.05 level.

Next, we hypothesized that continuous monochromatic light exposure would affect the levels of appetite genes in the hypothalamus, which plays an important role in appetite regulation, as well as in the maintenance of energy homeostasis in small nurse animals, and is the regulatory center of body weight and energy metabolism homeostasis. As illustrated in Figure 9B,D, the expressions of NPY and AgRP mRNA in the RL group were significantly higher than those in the GL and WL groups (*p* < 0.05). There was no significant difference in leptin mRNA levels between the three groups, and the expression of POMC mRNA in the RL group was lower than that in the WL and GL groups (*p* > 0.05). In addition, RL treatment also significantly increased the expression level of ghrelin in the hypothalamus (*p* < 0.05).

## 4. Discussion

Our study examined the effects of prolonged exposure to different colors of light on appetite, dietary preference, and glycolipid metabolism in mice fed both high-fat and low-fat foods. Our data show that the illumination color did not affect the dietary preferences of the mice, which chose high-fat foods in all three light environments and consumed very little low-fat food. This is similar to the results of Yang’s study, in which all rats chose high-protein, high-fat foods when allowed to choose their food [29]. This is contrary to the findings of Anayanci et al. This may be because their experiments selected a diurnal rodent, *Arvicanthis ansorgei* [30], whereas the present experiment used nocturnal rodents. Only the red light group further increased the body weight of the mice compared with the WL and GL groups. This was confirmed by their food intake, where mice in the RL group had the highest intake of high-fat chow and a significantly higher calorie intake than those in the WL and GL groups during the light period. Epididymal fat and abdominal fat are typical white fats, and studies have shown that an increased fat content (especially white fat content) is a key factor in obesity [31]. The H&E staining results also discovered that mice exposed to red light strongly triggered fat accumulation in the liver. Mice in the red light group had significantly higher levels of these two fats than those in the other two groups (*p* < 0.05), which may explain the relatively higher body weight of the mice in the red light group. In addition, it has been shown that mice prefer warm-light cages and have higher activity levels under red light [9].

To date, several studies have revealed the physiological and metabolic effects of exposure to specific colors of light in humans and food-borne animals [2,32,33]. For example, it was found that green light exposure exacerbated hepatic steatosis and pancreatic dysfunction induced by high-fat diet feeding in male mice. Our study found that prolonged exposure to different colors of light exacerbated metabolic disorders, including weight gain and dyslipidemia, in high-fat-fed mice. Meanwhile, GL and RL increased hyperlipidemia, as manifested by elevated serum and liver lipid levels. Experiments have shown that small cells irradiated with infrared light are less tolerant to glucose [27]. Guan’s study found that exposure to monochromatic blue light resulted in weight gain, obesity, and hepatic lipid degeneration [12]. Zhang et al.‘s study found that green light exposure aggravated high-fat-diet-induced hepatic steatosis and pancreatic dysfunction in male mice [2]. Different research methods may lead to different findings, and the main reason may be the differences in light intensity, exposure time, and wavelength. Changes in these parameters have important effects on the metabolism and physiology of the organism [34]. The illumination time in this study was during the daytime; however, mice are nocturnal animals, and most of their feeding and activity takes place at night, so our illumination time was nighttime. As we found, long-term exposure to RL led to dyslipidemia in mice. These symptoms were particularly significant in the RL-exposed group of mice, as evidenced by the persistently elevated blood glucose levels. Our research powerfully demonstrated that besides nutrient signaling, illumination color exerts a far-reaching role in the regulation of peripheral metabolic processes. It has a wide-ranging effect on the control of peripheral metabolic processes.

In the present study, we discovered that exposure to RL disrupted glucose homeostasis in mice. Cortical glial cells in the brain were found to synthesize and secrete insulin, and insulin levels in the brain were higher than those in the plasma [35]. In the hypothalamus, insulin is involved in the regulation of glucose homeostasis, central glucose transport, appetite, and metabolism [36]. It has been found that a high-fat diet increases levels of cholesterol and triglycerides, which can interact with insulin secreted by beta cells and inhibit insulin release [37,38]. The blood glucose output to the blood was higher in mice receiving RL irradiation than in the RL and WL groups. Moreover, in the OGTT experiment, the blood glucose values of mice in the RL group were higher than those in the other two groups after 2 h of metabolism, which may have been achieved by inhibiting insulin release, consistent with the description of Opperhuizen’s study [10].

As illustrated in Figure 8, mice in the RL group had the lowest leptin content, and the levels of appetite, hunger, and NPY hormones were significantly higher than those in the GL and WL groups. Many existing studies have shown that neuropeptides such as hypothalamic neuropeptide Y (NPY), orexin, orexin, agonist-associated protein (AgRP), and opioid adrenocorticotropic hormone progenitor the anorectic neuropeptides pro-opiomelanocortin (POMC) are involved in hypothalamic neural circuits to regulate ingestive behavior and energy homeostasis [39,40]. Mice exposed to red light had the highest levels of the hunger hormone and orexin. The qPCR analysis showed that RL upregulated NPY and AGRP mRNA levels and significantly decreased POMC mRNA levels. Peripheral tissue-derived insulin [41] and leptin [42] inhibited NPY and AGRP expression in the arcuate nucleus, promoted POMC expression, and reduced appetite, while the gastric starvation hormone had the opposite effect [43,44,45]. In addition, it has been demonstrated that hyperlipidemia leads to increased NPY secretion in the central nervous system of the brain, which is also in agreement with our findings. This also implies that NPY, AGRP, and POMC have a significant role in the regulation of food intake under different illumination environments. Therefore, it is reasonable to speculate that RL may regulate the expression of appetite genes in the hypothalamus by activating the secretion of pro-appetite neuropeptides from neurons in the central amygdala region [46], which increases appetite, promotes fat accumulation, and worsens energy metabolism [47], to influence eating behavior and dietary preferences.

Photosensitive retinal ganglion cells (ipRGCs) respond to light with opsin nigra (OPN4), so humans, mice, and rodents lacking optic rod and cone cells can perceive light [48,49]. Walmsley et al. demonstrated that illumination color can reach SCN neurons via optic vertebrae cell inputs from optic protein co-expression and, thus, affect circadian rhythms [50]. It has been observed that the hypothalamic tract (GHT) responds to light and actively regulates the SCN, which, in turn, regulates metabolism [51]. These studies indicate that different colors of light may exert different effects on the suprachiasmatic nucleus (SCN) and other brain regions. Our study showed that RL disrupted glycolipid metabolism in mice, possibly regulated by this pathway. ipRGCs are essential for non-image-forming visual functions of light. Lateral habenula (LHb) postsynaptic neurons regulate emotions by projecting to the DRN and VTA, like food rewards [52]. RL causes anxiety in mice, and they tend to crave high-fat and high-sugar foods [53,54,55]. This could also explain our findings in the present study.

Our study had the limitation of not considering the impact of illumination color on circadian rhythms, which could influence both appetite and metabolism in mice [56,57]. Further studies on hypothalamic neurons, synaptic function, and the role of skeletal muscle in glucose homeostasis are also needed to more deeply assess the effects of illumination color on appetite and glucose–lipid metabolism [58].

## 5. Conclusions

Taken together, our study demonstrates that exposure to RL promotes obesity in HDF-fed mice, leading to glucose intolerance, which may be due to light-induced changes in appetite hormone and insulin levels regulating appetite gene expression. Specifically, our findings provide additional insights into the relationship between light, appetite, and metabolic disorders. The effects of the lighting environment on metabolic homeostasis and appetite can be further investigated in depth through population or clinical trials.

## Figures and Tables

**Table 1:** The ingredients and energy levels of the diets.

Raw Materials	D12450J (10 kcal% Fat)		D12492 (60 kcal% Fat)	
	gm	kcal	gm	kcal
Casein	200	800	200	800
L-cystine	3	12	3	12
Corn starch	506.2	2024.8	0	0
Maltodextrin 10	125	500	125	500
Sucrose	68.8	275.2	68.8	275.5
Cellulose, BW200	50	0	50	0
Soybean oil	25	225	25	225
Lard	20	180	245	2205
Mineral Mx S10026	10	0	10	0
Dicalcium phosphate	13	0	13	0
Calcium carbonate	5.5	0	5.5	0
Potassium citrate, 1 H_2_O	16.5	0	16.5	0
Vitamin Mx V10001	10	40	10	40
Choline bitartrate	2	0	2	0
FD&C Yellow Dye #5	0.04	0	0	0
FD&C Blue Dye #1	0.01	0	0.05	0
	Gm%	Kcal%	Gm%	Kcal%
Protein	19.2	20	26.2	20
Carbohydrate	67.3	70	26.3	20.1
Fat	4.3	10	34.9	59.9
Total		100		100
Total	1055.05	4057		4057

**Table 2:** The information on the primers used for the PCR.

Item		Base Sequence (5′-3′)
Ghrelin	Forward	CATCCCCAGGCATTCCAGGTC
	Reverse	TCGAAGGGAGCATTGAACCTGAT
Leptin	Forward	GGAATTCAGGAAAATGTGCTGGAGA
	Reverse	GGAATTCTCAGCATTCAGGGCTAAC
NPY	Forward	CGCTCTGCGACACTACATCA
	Reverse	AGGGTCTTCAAGCCTTGTTCT
AgRP	Forward	AGAGTTCTCAGGTCTAAGTCT
	Reverse	CTTGAAGAAGCGGCAGTAGCACGT
POMC	Forward	CAGCGAGAGGTCGAGTTTG
	Reverse	CTGCTTCAGACCTCCATAGATGTG
GAPDH	Forward	CAAGGAGTAAGAAACCCTGGACC,
	Reverse	CGAGTTGGGATAGGGCCTCT

**Table 3:** Circulating lipid levels in mice after 8-week interventions of HFD feeding and light exposure (n = 14).

Parameters	RL	GL	WL
Serum TC (mmol/L)	3.15 ± 0.09 ^a^	2.26 ± 0.21 ^b^	2.10 ± 0.16 ^b^
Serum TGs (mmol/L)	5.78 ± 0.14 ^a^	2.76 ± 0.42 ^c^	3.14 ± 0.16 ^b^
Serum LDL-C (mmol/L)	3.11 ± 0.33 ^a^	1.79 ± 0.33 ^b^	1.45 ± 0.39 ^c^
Serum HDL-C (mmol/L)	1.55 ± 0.18 ^b^	1.68 ± 0.26 ^b^	1.78 ± 0.26 ^a^
Serum LDL-C/HDL-C ratio	2.03 ± 0.26 ^a^	1.08 ± 0.23 ^b^	0.83 ± 0.22 ^c^

Note: Means with different lower-case letters (a–c) within one row differed significantly between groups at *p* < 0.05.

## Data Availability

The data will be made available upon request.
